# Molecular Characterization of Resistance to Nicosulfuron in *Setaria viridis*

**DOI:** 10.3390/ijms24087105

**Published:** 2023-04-12

**Authors:** Yi Cao, Yuning Lan, Hongjuan Huang, Shouhui Wei, Xiangju Li, Ying Sun, Ruolin Wang, Zhaofeng Huang

**Affiliations:** State Key Laboratory for Biology of Plant Diseases and Insect Pests, Institute of Plant Protection, Chinese Academy of Agricultural Sciences, Beijing 100193, China

**Keywords:** acetolactate synthase, cytochrome P450, metabolic resistance, RNA sequencing

## Abstract

The green foxtail, *Setaria viridis* (L.) P. Beauv. (Poales: Poaceae), is a troublesome and widespread grass weed in China. The acetolactate synthase (ALS)-inhibiting herbicide nicosulfuron has been intensively used to manage *S. viridis*, and this has substantially increased the selection pressure. Here we confirmed a 35.8-fold resistance to nicosulfuron in an *S. viridis* population (R376 population) from China and characterized the resistance mechanism. Molecular analyses revealed an Asp-376-Glu mutation of the *ALS* gene in the R376 population. The participation of metabolic resistance in the R376 population was proved by cytochrome P450 monooxygenases (P450) inhibitor pre-treatment and metabolism experiments. To further elucidate the mechanism of metabolic resistance, eighteen genes that could be related to the metabolism of nicosulfuron were obtained bythe RNA sequencing. The results of quantitative real-time PCR validation indicated that three ATP-binding cassette (ABC) transporters (*ABE2*, *ABC15*, and *ABC15-2*), four P450 (*C76C2*, *CYOS*, *C78A5*, and *C81Q32*), and two UDP-glucosyltransferase (UGT) (*UGT13248* and *UGT73C3*), and one glutathione S-transferases (GST) (*GST3*) were the major candidates that contributed to metabolic nicosulfuron resistance in *S. viridis*. However, the specific role of these ten genes in metabolic resistance requires more research. Collectively, *ALS* gene mutations and enhanced metabolism may be responsible for the resistance of R376 to nicosulfuron.

## 1. Introduction

Green foxtail (*Setaria viridis* (L.) P. Beauv. (Poales: Poaceae)) is a diploid and self-pollinating grass weed. Its fecundity is high, and a single plant can produce more than 80,000 seeds; furthermore, *S. viridis* infests corn, millet, watermelon, and soybeans, leading to considerable yield losses [1]. *Setaria. viridis* is the second-largest weed in maize fields in Northeast China [2]. The control of *S. viridis* in maize fields has relied on the extensive use of the acetolactate synthase (ALS) inhibitor nicosulfuron during the last 20 years. Nicosulfuron belongs to the sulfonylurea group of ALS inhibitors that reduce branched-chain amino acid synthesis, which inhibits ALS enzyme activity in sensitive plants [3]. However, with the extensive use of nicosulfuron, some *S. viridis* populations have developed different levels of resistance in China [4,5].

Mechanisms of herbicide resistance include the following two types: target-site resistance (TSR) and non-target-site resistance (NTSR). To date, most cases of herbicide resistance reported have occurred through TSR, by which the herbicide binding site is mutated or the herbicide target gene is overexpressed [6]. For ALS-inhibiting herbicide resistance, various amino acid substitutions conferring resistance were identified at the eight main sites of Ala122, Pro197, Ala205, Asp376, Arg377, Trp574, Ser653, and Gly654 in the ALS isozymes of weeds. Mutations in the *ALS* gene confer resistance to many weeds, such as *Solanum ptychanthum* Dunal (Solanales: Solanaceae) [7], *Amaranthus palmeri* S.Watson (Caryophyllales: Amaranthaceae) [8], *Erigeron sumatrensis* Retz. (Asterales: Asteraceae) [9], *Glebionis coronaria* (Linnaeus) Cassini ex Spach (Asterales: Asteraceae) [10], etc.

Compared with TSR, NTSR is more complex, involving multiple genes. The NTSR may result from reduced herbicide penetration into the plant, herbicide translocation, reduced sequestration, or increased metabolic rate [6]. Enhancing the metabolism of herbicides in weeds is a current research hotspot. It involves cytochrome P450 monooxygenases (P450s), glucosyltransferases (GTs), ATP-binding cassette (ABC) transporters, and glutathione S-transferases (GSTs) [11]. The RNA sequencing (RNA-Seq) technology efficiently quantifies gene expression through short-read sequencing on the Illumina platform. Genes conferring NTSR resistance in weeds were identified by this technique [12]. For example, *CYP72A31* confers tolerance to ALS-inhibiting herbicides in rice and *Arabidopsis thaliana* (L.) Heynh. (Capparales: Brassicaceae) [13]. In addition, *CYP749A16*, *CYP71C6v1*, and the P450 genes belonging to the *CYP81A* subfamily are related to the metabolism of ALS-inhibiting herbicides [13,14,15].

The TSR and NTSR mechanisms of weeds can be superimposed in the same population or the same plant [16,17], such as *Lolium rigidum* Gaudich. (Poales: Poaceae) [18,19], *Amaranthus tuberculatus* (Moq.) J. D. Sauer (Caryophyllales, Amaranthaceae) [20], and *S. viridis* [5]. The mechanisms that confer herbicide resistance might have effects on the weed growth. For example, the *S. viridis* mutants resistant to acetyl-CoA carboxylase (ACCase) herbicides showed better growth and a higher number of grains than the wild type [21]. Additionally, the *S. viridis* resistant to dinitroaniline were smaller and had a lower thousand-grain weight compared with the wild type [22]. *S. viridis* is the wild ancestor of *Setaria italica* (L.) Beauv. (Poales: Poaceae) and exhibits an evolutionary relationship with maize, sorghum, and sugarcane [23,24]. Therefore, studying the resistance mechanism in *S. viridis* is of great significance for weed control and crop breeding. In recent years, maize growers from Northeast China have complained about unsatisfactory control of this weed after nicosulfuron treatment. The objectives of this study are to (1) identify the target-site basis of the possible presence of a point mutation in the *ALS* gene and (2) determine the NTSR and identify candidate genes in *S. viridis*.

## 2. Results

### 2.1. Identification of Resistance-Associated Mutations in ALS

**Figure 1:**
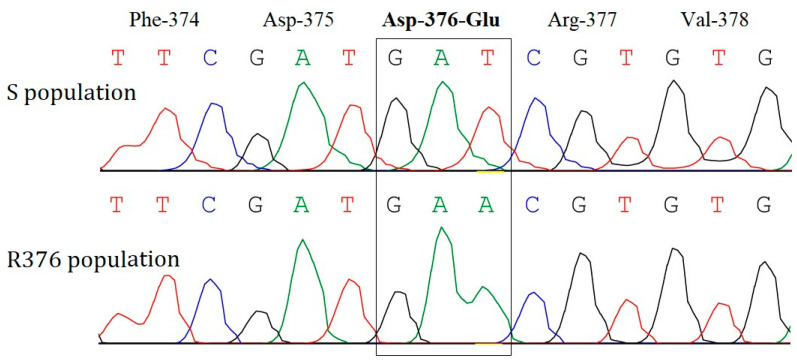
Comparison of *acetolactate synthase* (*ALS*) gene partial sequences from S and R376 populations of *Setaria viridis*.

To determine whether mutations in the *ALS* gene led to nicosulfuron resistance in *S. viridis*, ten plants were randomly selected and *ALS* sequenced from each of the suspected sensitive (S) and resistant (R376) populations. The *ALS* gene fragment of *S. viridis* spanning all known mutations was sequenced. The analysis of the *ALS* sequence of the S and R376 populations showed that no known or novel mutations were detected in the S population, but a codon shifted from GAT to GAA, resulting in an Asp-376-Glu substitution that was detected in the R376 population (Figure 1).

### 2.2. Effect of Pre-Treatment with Malathion on Nicosulfuron Resistance

**Figure 2:**
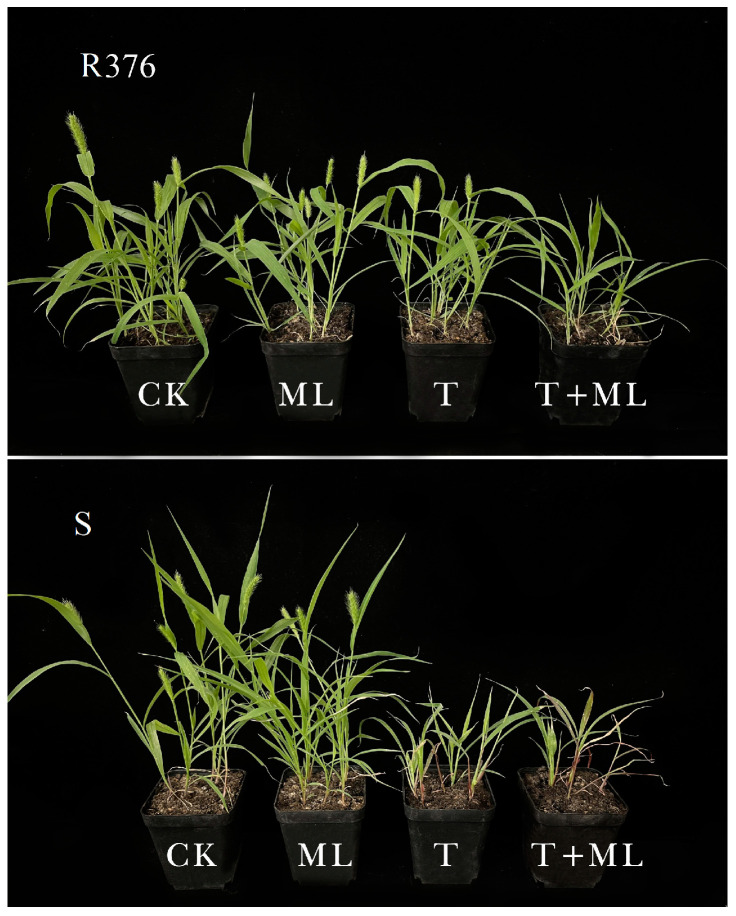
Effects of malathion pre-treatment in S and R376 populations of *S. viridis*. CK, ML, T, and T + ML represent the treatment of water, malathion, nicosulfuron (30 g a.i. ha^−1^), and malathion + nicosulfuron (30 g a.i. ha^−1^).

The whole-plant dose–response results showed that the GR_50_ values of the R376 and S populations to nicosulfuron were 268.6 and 7.5 g a.i. ha^−1^, respectively, which indicated that the R376 population is 35.8-fold more resistant to nicosulfuron than the S population. Malathion pre-treatment with nicosulfuron resulted in greater injury to the R376 population and the GR_50_ was 159.8 g a.i. ha^−1^, whereas the GR_50_ of the S population did not change significantly (Table 1, Figure 2). Therefore, the treatment of malathion plus nicosulfuron reduced the resistance factor of the R376 population by 37.2%.

### 2.3. Nicosulfuron Metabolism in S. viridis Plants

**Figure 3:**
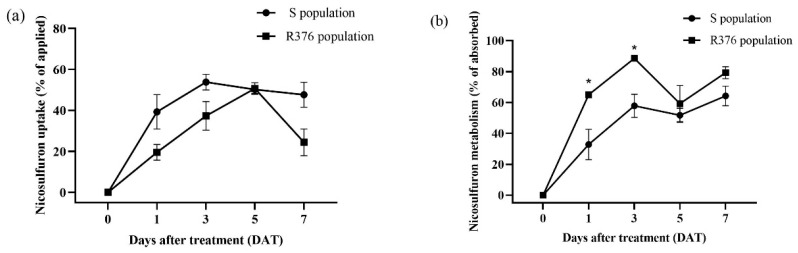
Nicosulfuron absorption (**a**) and metabolism (**b**) in S and R376 populations of *S. viridis*. * *p* < 0.05.

In this experiment, the retention time of nicosulfuron was 2.72 min, and the average recoveries were 95–110%. At 1, 3, and 7 days after treatment (DAT), the absorption of nicosulfuron in the S population was higher than that in the R376 population. However, there was no statistically significant difference between the two groups. The metabolism rate of nicosulfuron in the R376 population was faster than that in the S population. At the same time, statistical analysis showed that the metabolic ability of nicosulfuron in the R376 population was significantly higher than that in the S population at 1 and 3 DAT (Figure 3).

### 2.4. Transcriptome Sequencing and Assembly

The RNA-Seq was performed on 12 samples, each with >5.89 GB of clean data. A total of 78.31 Gb of clean data was obtained in the experiment, and more than 92% reached the Q30 (Table 2). The data were compared with the internal reference gene of *S. viridis*, and the consistency rate was greater than 93%.

A total of 38,986 genes were annotated throughout transcriptome sequencing using the databases, Nr (NCBI non-redundant protein sequence), Nt (NCBI non-redundant nucleotide sequence), Pfam (Protein family), KOG/COG (Clusters of Orthologous Groups of proteins), Swiss-Prot (manually annotated and reviewed protein sequence database), KO (KEGG Orthologue database) and GO (Gene Ontology) databases (Table 3). A comparison of gene annotation results showed that the experimental *S. viridis* was most similar to the model *S. viridis* (74.62% of total unigenes) and *S. italica* (24.42%). This result showed that the transcriptome data were reliable.

### 2.5. Selection of Candidate Genes

We identified 1691 differentially expressed genes (DEGs) between the RT and ST groups (974 upregulated and 717 upregulated) and 5339 DEGs between the RT and RC groups (2172 upregulated and 3167 upregulated). Compared with the SC group, 1056 upregulated genes were found in the RC group and 3568 genes were upregulated in the ST group (Table 4).

As the R376 population more rapidly metabolizes nicosulfuron in comparison with the S population, we focused on the analysis of DEGs associated with nicosulfuron metabolic resistance. By screening annotations related to metabolism, 11 DEGs were identified. The DEGs identified in the differential expression analysis were annotated, and a summary of the annotations is shown in Table 5. Among them, two genes (*6G242300*, *9G351200*) belonged to the GST family, four genes (*6G098000*, *9G011000*, *8G128400*, *9G081600*) were annotated to the P450 family, two genes (*7G025600*, *4G081200*) encoded UGTs, and four genes (*1G127100*, *7G083400*, *7G083600*) belonged to the ABC transporter family (Table 5).

**Figure 4:**
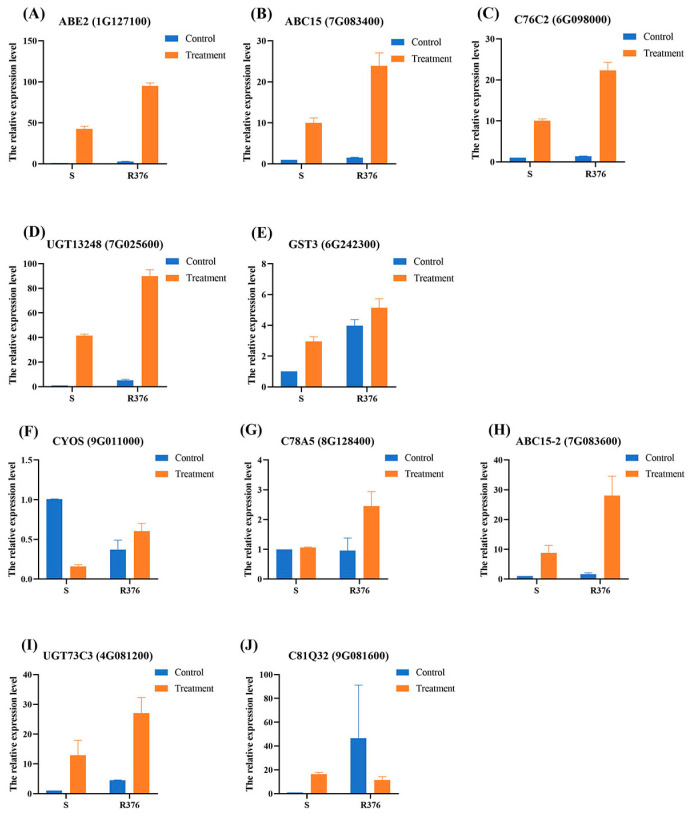
Relative expression of ten candidate genes in S and R376 populations before (Control) and after (Treatment) nicosulfuron treatment. The ten candidate genes are *ABE2* (**A**), *ABC15* (**B**), *C76C2* (**C**), *UGT13248* (**D**), *GST3* (**E**), *CYOS* (**F**), *C78A5* (**G**), *ABC15-2* (**H**), *UGT73C3* (**I**), *C81Q32* (**J**).

The expression levels of candidate genes were verified by quantitative real-time PCR (qPCR). According to the results, the candidate genes were divided into three groups. Five genes (*ABE2*, *ABC15*, *C76C2*, *UGT13248*, and *GST3*) were grouped into one group (Figure 4A–E). In this group, regardless of whether nicosulfuron was applied or not, the expression levels of these five genes in the R376 population were significantly higher than those in the S population. For example, the expression of the *ABE2* gene in R376 control and treatment groups was significantly higher than that in S, 2.9-fold and 2.3-fold, respectively (Figure 4A). The genes in the second group were *CYOS*, *C78A5*, *ABC15-2*, and *GSTU6* (Figure 4F–H). In the control group, the expression levels of the four genes in the R376 population were lower than those in the S population or had no significant difference, but after herbicide treatment, their expression levels in R376 were significantly higher than those in the S population, except for *GSTU6*. The candidate genes in the last group (*UGT73C3* and *C81Q32*) were significantly upregulated in RC vs. SC but not in RT vs. ST (Figure 4I,J). Therefore, three ABC transporters (*ABE2*, *ABC15*, and *ABC15-2*), four P450s (*C76C2*, *CYOS*, *C78A5*, and *C81Q32*), two UGTs (*UGT13248* and *UGT73C3*), and one GST (*GST3*) could serve as the main candidate genes for metabolic resistance to nicosulfuron.

## 3. Discussion

*Setaria viridis* is one of the most common gramineous weeds in spring maize fields in northern China that seriously endangers maize yield and is the focus of control [2]. Nicosulfuron has been used in maize fields in China for more than 20 years. Due to its high activity, strong selectivity, low residue, and small impact on subsequent crops, it has gradually developed into an extensively used herbicide for controlling gramineous weeds in maize fields in China. The consecutive use of nicosulfuron has resulted in the evolution of resistance in *S. viridis*. In this study, the GR_50_ of the R376 population was 4.48-fold higher than the recommended field dose and 35.8-fold higher than that of the S population, which indicated that a high dose of nicosulfuron could not control *S. viridis* and that the R376 population was resistant to nicosulfuron.

The TSR is the main cause of resistance to herbicides. Analysis of the *ALS* sequences of R376 plants indicated the presence of the Asp-376-Glu substitution. The Asp376 substitution results in high levels of resistance to sulfonylurea (SU) herbicides in *Schoenoplectiella juncoides* (Roxb.) Lye (Poales: Cyperaceae)*, Lolium perenne* L. (Poales: Poaceae)*, Limnocharis flava* (L.) Buchenau (Alismatales: Alismataceae)*,* etc. [25,26,27]. This result indicated that targeted mutation was an important reason for the resistance of the R376 population to nicosulfuron.

All types of resistance mechanisms, including TSR and NTSR, can be superimposed in resistant plants. In a previous study, *S. viridis* populations with high resistance to nicosulfuron were shown to possess both target-site mutations (Asp-376-Glu and Pro-197-Ala) and P450-mediated enhanced metabolism [5]. Metabolic resistance in weeds first became evident in the 1980s in Australia (in *L. rigidum*) and the United Kingdom (in *Alopecurus myosuroides* Huds. (Poales: Poaceae)), and this type of resistance is now increasing in weeds [17]. In our study, the increased susceptibility to nicosulfuron in the R376 population after malathion pre-treatment suggested possible metabolism-based resistance in R376. To obtain more direct evidence of metabolic resistance to nicosulfuron in R376, differences in the metabolism of nicosulfuron in the S and R376 populations were analysed by HPLC-MS, and the results showed that the R376 population metabolized nicosulfuron more strongly than the S population. These results support the hypothesis that metabolic resistance played a crucial role in nicosulfuron resistance in the R376 population.

The RNA-Seq transcriptome analysis has been used to study metabolic herbicide resistance in weeds for gene discovery. Increased activity of endogenous P450s, GTs, GSTs, and/or other enzyme systems confers the ability to metabolize herbicides [28]. In our study, a total of four P450 genes, two GST genes, three ABC transferases, and two UGT were significantly upregulated in the R376 population compared with the S population.

The P450 genes can metabolize herbicides through alkyl-hydroxylation, N-demethylation, O-demethylation, and aryl-hydroxylation. More than 30 P450 genes have been reported to metabolize herbicides, 11 of which can metabolize ALS-inhibiting herbicides. Among plant families, Poaceae has the largest number of herbicide metabolism-related genes reported [29]. The CYP81A12 and CYP81A21 proteins metabolize bensulfuron-methyl through O-demethylation and confer resistance to two classes of ALS-inhibiting herbicides in *Echinochloa phyllopogon* (Stapf) Koss (Poaceae: Panicaceae) [15]. Upregulation of *CYP709C56* in *Alopecurus equalis* Sobol. (Poales: Poaceae) detoxifies metsulfuron-methyl to an O-demethylated metabolite, thereby conferring resistance [30]. In the present study, *C76C2*, *C81Q32*, *CYOS*, and *C78A5* were identified, but their specific role in metabolic resistance to nicosulfuron requires further investigation. The GSTs are multifunctional enzymes involved in the normal metabolism of endogenous substrates and phase II metabolic detoxification of exogenous substances, which play an important role in defending against pathogens [31,32]. In our study, *GST3* and *GSTU6* were identified as candidate genes. It has been shown that high-level expression of *GSTT3* and *GSTU6* can confer resistance to fenoxaprop-P-ethyl and mesosulfuron-methyl in *Beckmannia sizigachne* (Steud.) Fernald (Poales: Poaceae) [33].

By compartmentalizing herbicides and their metabolites, ABC transporters give herbicide resistance in contrast to the P450 and GST gene families; this procedure is comparable to phase III detoxification [11]. Glyphosate resistance may be conferred when tonoplast or PM ABC transporters can transfer the herbicide out of the cytoplasm [34]. The expression of *AB2E*, *ABC15*, and *ABC15-2* was increased in the R376 population compared with the S population. In addition to being involved in the detoxification process, the ABC transporters also play an important role in the growth and development of the plant, for example, in chlorophyll biosynthesis, the formation of Fe/S clusters, stomatal movement, and probably ion flux [35,36]. Further research is required to fully describe the functional characterization of the candidate genes identified in this study.

In plants, UGT can glycosylate phytohormones, secondary metabolites involved in stress and defense responses, and xenobiotics such as herbicides, altering their properties [37]. A lower *UGT13248* expression was detected in the S population before and after nicosulfuron treatment in contrast with the R376 population. In the control group, the expression level of *UGT73C3* was significantly higher in R376 than in the S population. Further experiments are needed to investigate whether *UGT13248* and *UGT73C3* can glycosylate nicosulfuron.

## 4. Materials and Methods

### 4.1. Plant Materials and Growth Conditions

The putative-resistant *S. viridis* population was collected randomly from maize fields in Jilin Province in China, where nicosulfuron had been continuously applied for more than 10 years. The susceptible *S. viridis* population (S) was obtained from non-cultivated areas (Tongyu, China). The seeds of each population were harvested from more than thirty plants.

After cleansing with water, *S. viridis* seeds were submerged in an 800 mg L^−1^ solution of gibberellin (GA_3_) for 24 h. The germinated seeds, twenty seeds per pot, were evenly planted in plastic pots with an 11-cm diameter, and they were nurtured in a greenhouse (day and night, 30/22 °C, 14 h/10 h photoperiod). The seedlings were thinned to 10 plants per pot at the 2-leaf stage, watered every two days, and fertilized weekly. The *S. viridis* seedlings can be used for experiments at the 3–4 leaf stage.

### 4.2. Sequencing of the ALS Gene

Leaves from the R376 population that survived nicosulfuron (60 g a.i. ha^−1^) treatment and S population that untreated were harvested. Leaves of ten plants were collected from each population of the untreated S and R376, and genomic DNA was extracted by the DANsecure Plant Kit^®^ (Tiangen Biotechnology Co., Ltd., Beijing, China). Then a partial *ALS* gene containing all eight known site mutations was amplified according to Huang et al. [5]. The PCR products were confirmed on a 1% agarose gel and sequenced by the Beijing Genome Institute (BGI, Beijing, China). Sequence data were aligned and analyzed using Vector NTI 12.5 (Invitrogen Corporation, Carlsbad, CA, USA).

### 4.3. Effect of Malathion on Nicosulfuron Resistance

When *S. viridis* seedlings were at the 3–4 leaf stage, a whole-plant dose−response assay was conducted to determine resistance to nicosulfuron (40 g L^−1^ OD, Beijing Green Agricultural Science and Technology Group Co., Ltd., Beijing, China) with or without malathion (70% EC, Dezhou Luba Fine Chemical Co., Ltd., Dezhou, China). Nicosulfuron and malathion were applied using a research track sprayer (3 WP-2000) (Nanjing Institute of Agricultural Mechanization, Ministry of Agriculture and Rural Affairs, China) delivering 450 L ha^−1^ spray solution. Malathion, a common P450 indicator for metabolic resistance, was applied at 2000 g a.i. ha^−1^ one hour before nicosulfuron application. Preliminary assays showed that there was a large difference in the sensitivity to nicosulfuron between S and R376 populations, so nicosulfuron was applied at 0, 3.75, 7.5, 15, 30, 60, and 120 g a.i. ha^−1^ for the S population and at 0, 30, 60, 120, 240, 480, and 960 g a.i. ha^−1^ for the R376 population. Three weeks after treatment, the aboveground parts of the plants were harvested and the fresh weight was recorded. The experiment was designed as a randomized complete block with three pots in each replicate, and the experiment was repeated twice.

The mean fresh weight for each treatment was expressed as a percentage of the untreated control. The data were subjected to non-linear regression analysis using the following formula to obtain dose–response curves (SigmaPlot, San Jose, CA, USA).

Y = C + [(D − C)/[1 + (X/GR_50_)^b^] (1)

where C is the lower limit, D is the upper limit, and b is the relative slope around the herbicide dose causing a 50% growth reduction (GR_50_).

### 4.4. HPLC-MS/MS Analysis of Nicosulfuron

When *S. viridis* seedlings were at the 3–4 leaf stage, nicosulfuron (5 μL of 1 mg L^−1^ nicosulfuron per plant) was added dropwise on leaves using micropipettes. The plants from both the R376 and S populations were randomly selected at 1, 3, 5, and 7 DAT. Plants were washed with 10 mL of acetonitrile and stored at −20 °C. The solution (1 mL) was placed in a vial for absorption analysis.

Samples were ground in liquid nitrogen and placed in 50 mL centrifuge tubes containing 10 mL of 0.1% formic acid in acetonitrile. Then they were vortexed for 5 min and sonicated for 10 min. After centrifugation at 6000 rpm for 10 min, 1.5 mL of the supernatant sample was transferred to a 5 mL centrifuge tube containing 20 mg Graphitized carbon black (GCB) (Agela Technologies, Tianjin, China), 100 mg MgSO_4_ (Sinopharm Chemical Reagent Co., Ltd., Shanghai, China), and 20 mg ethylenediamine-N-propylsilane (PSA) (Agela Technologies). The 5 mL centrifuge tubes were then vortexed for 1 min and centrifuged at 12,000 rpm for 5 min. An amount of 1 mL of the supernatant passed through a 0.22 µm filter and transferred to a 2 mL autosampler vial for detection. Nicosulfuron was analysed using AB SCIEX QTRAP 5500 HPLC-MS/MS (AB Sciex, Concord, ON, Canada) and the chromatographic column was ACQUITY UPLC C18 column (2.1 mm × 100 mm, 1.7 µm) (Waters Corporation, Milford, MA, USA). The injection volume was 5 μL, and the whole HPLC run used 0.1% formic acid water (A) and acetonitrile (B) as the mobile phase at a flow rate of 0.3 mL/min. The mobile phase gradient decreased from 80% A to 40% A (0–1 min), then decreased to 20% A (1–2.5 min) and returned to 80% A (2.5–3 min). The scanning mode was positive ionization mode, and a mass-to-charge ratio of 182.2 as the quantifying ion. The absorption of nicosulfuron was measured as the total application minus the amount eluted from the plant surface; the metabolism was measured as the absorption minus the residue in the plant. The experiment was performed twice with three replicates for each treatment.

### 4.5. RNA Extraction, Library Construction, and Sequencing

An RNA-Seq experiment was conducted using the S and R376 populations. The samples included untreated S and R376 plants and S and R376 plants 24 h after treatment with nicosulfuron (the recommended field rate 60 g a.i. ha^−1^), with three biological replicates. The twelve samples were frozen immediately in liquid nitrogen, and total RNA was extracted using the RNAprep pure Plant Kit (Tiangen Biotech Co., Ltd., Beijing, China). Then, the high-quality RNA samples were transcribed into first-strand cDNA by random hexamer primers and M-MuLV reverse transcriptase. Second-strand cDNA synthesis was performed using DNA Polymerase I and RNase H. The quality of the cDNA library was assessed on the Agilent Bioanalyzer 2100 system and then sequenced on the Illumina Hiseq^TM^ 4000 platform (Illumina Inc., San Diego, CA, USA).

### 4.6. De Novo Transcriptome Assembly and Gene Function Annotation

In the experiment, sequencing data were analysed using the bioinformatics analysis process provided by BMKCloud (www.biocloud.net (accessed on 20 May 2021)) (BIOMARKER, Beijing, China). Firstly, the raw data were filtered to obtain high-quality clean data. These clean data were then further mapped to a reference genome to generate mapped data. After the quality assessment was qualified, analyses such as gene expression quantification, alternative splicing analysis, new gene prediction, and gene structure optimization were carried out. Quantification of gene expression levels was estimated by fragments per kilobase of transcript per million fragments mapped (FPKM).

### 4.7. Identification and Analysis of Differentially Expressed Genes

The DESeq2 was used to conduct a differential expression analysis of the two conditions/groups. The DEGs were defined as genes having a DESeq2 adjusted *p*-value < 0.01. The 12 samples were divided into the following four groups: treated S (ST), treated R376 (RT), untreated S (SC), and untreated R376 (RC). DEGs between RT and ST, RC and SC, RT and RC, and ST and SC were compared. Two points had to be satisfied when selecting candidate NTSR genes. Firstly, there had to be significant differential expressions (|log2(fold change)| > 1 and q (adjusted *p*-value) ≤ 0.05) between RT vs. ST, RC vs. SC. Secondly, the DEGs had to be involved in herbicide metabolism, such as the P450, GST, GT, and ABC transporters.

### 4.8. The qPCR Validation

The untreated S and R376 plants and the S and R376 plants 24 h after treatment with nicosulfuron (60 g a.i. ha^−1^) were collected for qPCR. Three biological replicates and three technical replicates were performed for each sample. The primers were designed in Primer Premier v.5.0 (Premier Biosoft International, Palo Alto, CA, USA) (Table 6). The qPCRs were performed at a final volume of 20 µL with the following reagents: 10 μL of 2× PerfectStart^®^ Green qPCR SuperMix (+DyeI/+DyeΠ) (TransGen Biotech company, Beijing, China), 0.4 μL of forwards primer, 0.4 μL of reverse primer, 1 μL of cDNA, and 8.2 μL of RNase-free ddH_2_O. All qPCR amplifications were performed using the following conditions: (1) 30 s at 94 °C for pre-denaturation; (2) 40 cycles of amplification (5 s at 94 °C for denaturation, 35 s at 60 °C for annealing and extension). Three technical replicates were performed for each experiment. All samples were normalized with the reference gene *serine/threonine protein phosphatase 2A* (*PP2A*) and calculated using the 2^−ΔΔCt^ method [38]. Statistical analysis of the qPCR data was conducted using the Student’s *t*-test (*p* < 0.05) by IBM SPSS Statistics version 24.0 (IBM Company, Chicago, IL, USA) software. The genes that have correlation with transcriptome and have significant differences (*p* < 0.05) are the target genes.

## 5. Conclusions

Generally, resistance resulting from NTSR occurs at relatively low levels and is difficult to detect. However, NTSR is more threatening than TSR because it may confer unpredictable resistance to herbicides with multiple modes of action, thus posing a great threat to agricultural production. We should further study the underlying mechanism of NTSR to provide a solid theoretical basis for the integrated management of herbicide-resistant populations of *S. viridis*.

## Figures and Tables

**Table 1:** Values of GR_50_ to nicosulfuron for S and R376 *S. viridis* populations with and without malathion. The coefficients of determination (R^2^) of four dose–response curves were > 0.99.

Treatments	S			R376			RI ^d^
	GR_50_ ^a^ (g a.i. ha^−1^) ± (SE) ^b^	95% CI ^c^	*p*	GR_50_ (g a.i. ha^−1^) ± (SE)	95% CI	*p*	
Nicosulfuron	7.5 ± 0.56	6.4–8.5	0.2075	268.6 ± 12.3	176.6–361.3	0.8014	35.8
Malathion + nicosulfuron	7.1 ± 0.81	5.7–9.0	0.0632	159.8 ± 5.9	93.4–226.2	0.4970	22.5

^a^ GR_50_, herbicide dose causing a 50% plant mortality. ^b^ SE, standard error. ^c^ CI, confidence intervals. ^d^ RI, resistance index = GR_50_ of R376 population/GR_50_ of S population.

**Table 2:** Sequencing data statistics.

Samples	Clean Reads ^a^	Clean Bases ^b^	GC Content ^c^	≥Q30% ^d^
Rck1	21,357,755	6,394,783,796	55.20%	92.56%
Rck2	20,859,879	6,247,437,338	54.66%	92.13%
Rck3	24,958,030	7,473,507,024	55.12%	92.19%
Rt1	20,964,984	6,275,572,248	53.72%	95.19%
Rt2	21,160,996	6,339,218,842	50.11%	93.73%
Rt3	21,802,295	6,530,031,944	53.52%	95.35%
Sck1	19,886,346	5,956,973,016	54.92%	95.40%
Sck2	25,714,276	7,700,027,284	54.97%	95.69%
Sck3	21,408,426	6,411,927,072	54.96%	95.59%
St1	22,129,252	6,625,605,236	53.42%	95.13%
St2	19,674,122	5,885,620,152	53.87%	95.27%
St3	21,613,683	6,467,906,952	54.01%	95.57%
Rck1	21,357,755	6,394,783,796	55.20%	92.56%

^a^ Clean reads: counts of clean PE reads; ^b^ clean bases: total base number of clean data; ^c^ GC content: percentage of G, C in clean data; ^d^ ≥Q30%: the percentage of bases with a Q-score is no less than Q30.

**Table 3:** Summary table of gene function annotations.

Annotation Database	Annotated Number	Annotated Percent (%)
COG	9863	24.5
GO	24,099	59.8
KEGG	20,174	50
KOG	14,738	36.5
Pfam	25,125	62.3
Swiss-Prot	20,508	50.9
eggNOG	25,648	63.6
NR	38,961	96.6
All	38,986	96.7

**Table 4:** The number of differentially expressed genes (DEGs) between different treatment groups.

Groups/Samples	DEG Number	Upregulated	Downregulated
RT vs. RC	5339	2172	3167
RC vs. SC	1582	1056	526
RT vs. ST	1691	974	717
ST vs. SC	8257	3568	4689

RT, resistant seedlings sprayed with nicosulfuron; ST, susceptible seedlings sprayed with nicosulfuron; RC, resistant seedlings without nicosulfuron; and SC, susceptible seedlings without nicosulfuron.

**Table 5:** The candidate differentially expressed genes related to non-target-site resistance of *S. viridis* to nicosulfuron.

GeneBank Accession Number	Log_2_ (RT/ST)		Log_2_ (RC/SC)		Gene Description
	RNA-Seq	qPCR	RNA-Seq	qPCR	
1G127100	2.21	1.16 ± 0.04 ***	1.63	1.54 ± 0.1 **	ABC transporter E family member 2
7G083400	3.39	1.25 ± 0.02 *	3.92	0.59 ± 0.08 *	ABC transporter C family member 15
6G098000	6.55	1.14 ± 0.05 *	3.66	0.46 ± 0.07 **	Cytochrome P450 76C2
7G025600	-	1.06 ± 1.22 ***	3.00	2.33 ± 0.18 *	UDP-glucosyltransferase UGT13248
6G242300	8.37	0.79 ± 0.11 *	7.13	1.97 ± 0.13 ***	Glutathione S-transferase T3
9G011000	3.31	1.92 ± 0.11 *	-	−1.68 ± 0.42 *	Premnaspirodiene oxygenase OS
8G128400	2.83	1.14 ± 0.20 *	-	−0.66 ± 0.70	Cytochrome P450 78A5
7G083600	1.16	1.79 ± 0.37 *	2.71	0.62 ± 0.27	ABC transporter C family member 15
4G081200	-	1.38 ± 0.50	3.99	2.14 ± 0.04 **	UDP-glycosyltransferase 73C3
9G081600	-	−0.62 ± 0.22	1.98	1.08 ± 0.12 *	Cytochrome P450 81Q32
9G351200	1.66	1.90 ± 0.16	-	−0.27 ± 0.08	Glutathione S-transferase GSTU6

* *p* < 0.05, ** *p* < 0.01, *** *p* < 0.001.

**Table 6:** Sequences of the primers used in this study.

Gene	Forward Primer (5′-3′)	Reverse Primer (5′-3′)
*PP2A*	ATGTGACACGGAGAACACCA	TGTTTCTGACCAGCAACCAC
1G127100	AGAACTCATGGACAGGCAAT	GGTAAATATCAGCAGGCTTTCCG
7G083400	CCCCAATGTCTTTCTTTGACTCC	GTCCCCAGGATTTGTATGACTGA
6G098000	AGCCATTCATTGAAGAGTCCGA	TGTAGCCGTTGACCTCGAT
7G025600	TTGGCACACAAGGCAACAGG	TGCCACCAACGGTACACCA
6G242300	AGGCCAAACCATAAGAGATCCAA	TTTGCCTACGACCGGATCCA
9G011000	TCAACATCCCGGACCTGT	CCGTCCTGTGGATGTCCT
8G128400	CAAGCCCGTAAAGCTTGCAG	TAAGCAGCTTCTCCACGACG
7G083600	CAAAGCGAAACGGGACTGTCA	ATTTCCGAATAGAATGTTGTCCCT
4G081200	CACCTTCGAGGAGATGGAGC	GCTGGTGGTAGAGTGACACC
9G081600	AGGCCAACGAGTTCAAGC	CGCCGAACACGTCGAACCA
9G351200	CTGAAGCTGCTCGGGATGTG	GCTCTTGTTGCGTAGGTTCTCC

## Data Availability

Readers are welcome to contact the corresponding authors if interested in using the data used in this study.
